# Vitamin C for sepsis: from mechanisms to individualized therapy

**DOI:** 10.3389/fmed.2025.1700351

**Published:** 2025-12-18

**Authors:** Yang Xiao, Fang Gong, Lina Zhang, Chunmei Gui

**Affiliations:** 1 Department of Intensive Care Unit, Changde Hospital, Xiangya School of Medicine, Central South University (The First People's Hospital of Changde City), Changde, China; 2 Department of Intensive Care Unit, Xiangya Hospital of Central South University, Changsha, China

**Keywords:** sepsis, septic shock, vitamin C, ascorbic acid, mechanism

## Abstract

Sepsis is a critical illness initiated by infection and characterized by a dysregulated inflammatory and oxidative stress response, leading to high mortality rates and impaired long-term quality of life. It is noteworthy that many sepsis patients have insufficient levels of vitamin C, an essential micronutrient. Due to its diverse physiological roles, including antioxidant, anti-inflammatory, immunomodulatory, and antimicrobial-enhancing effects, vitamin C has gained significant attention as a potential adjunctive therapy for sepsis. However, the specific mechanisms by which vitamin C acts in sepsis are still not fully understood. Recent preclinical studies have shown that it can help reduce sepsis-induced organ damage, but clinical trials assessing its effectiveness have produced mixed results. Importantly, vitamin C's pharmacological effects depend on its concentration, and it has complex pharmacokinetics, which makes establishing an appropriate dosage regimen critical for achieving therapeutic outcomes in patients. This review aims to synthesize the current evidence regarding the therapeutic mechanisms of vitamin C in sepsis, identify limitations in the existing clinical research, and highlight future directions for investigation.

## 1 Introduction

Sepsis, a life-threatening organ dysfunction caused by a dysregulated host response to infection, represents a major global public health concern, with an incidence of 677.5 cases per 100,000 people and a mortality rate of 148.1 per 100,000—accounting for 19.7% of global deaths (1). The primary pathological mechanism of sepsis-induced organ injury involves microcirculatory dysfunction, in which the endothelial glycocalyx—a critical layer of negatively charged polysaccharides and proteins essential for maintaining microvascular homeostasis—plays an important role. This structure regulates vascular tone and permeability while inhibiting leukocyte and platelet adhesion to the endothelium. During sepsis, activation of Toll-like receptors by pathogen- and damage-associated molecular patterns triggers dysregulated inflammation and oxidative stress. This cascade disrupts glycocalyx structural integrity and induces endothelial damage, exacerbating capillary leakage, microvascular immunothrombosis, and microcirculatory failure, ultimately leading to multiorgan dysfunction (2–5).

Vitamin C (ascorbic acid) is an essential micronutrient, and most sepsis patients present with insufficiency, primarily due to metabolic consumption (6–9). Despite receiving standard nutritional support in the ICU, 88% of septic shock patients developed hypovitaminosis C (plasma concentration <23 μM), with 38% falling into the criteria for severe deficiency (<11 μM) (6). Importantly, initial vitamin C deficiency was significantly associated with increased 28-day mortality risk in patients with septic shock (adjusted RR: 2.65, 95% CI: 1.08–6.52, *P* = 0.032) (10), and lower vitamin C levels were also associated with more severe disease in children with sepsis (8). However, the effectiveness of vitamin C treatment regimens, whether administered alone or in combination with other agents, in improving organ function and mortality in sepsis patients remains a subject of debate (11–13). Several recent cohort studies have suggested that moderate-dose, longer-duration vitamin C regimens may significantly reduce mortality in patients with sepsis (14–16).

Functioning as an electron donor, vitamin C exhibits concentration-dependent redox effects (17). At concentrations ranging from 1 to 100 μM, it better protected endothelial function and improved microcirculation by increasing tetrahydrobiopterin (BH4) levels, a coenzyme of endothelial nitric oxide synthase (eNOS). However, concentrations exceeding 1 mM may produce pro-oxidant effects that interfere with BH4 elevation, underscoring the importance of an appropriate dosage regimen (18, 19).

Considering the substantial burden of sepsis, the widespread vitamin C insufficiency among patients, and the ongoing controversies regarding clinical outcomes, this review aims to elucidate the molecular mechanisms of vitamin C-mediated organ protection. Additionally, it will critically assess the limitations and controversies present in current clinical research and identify key directions for future investigation.

## 2 Organ-protective mechanisms of vitamin C

**Figure 1:**
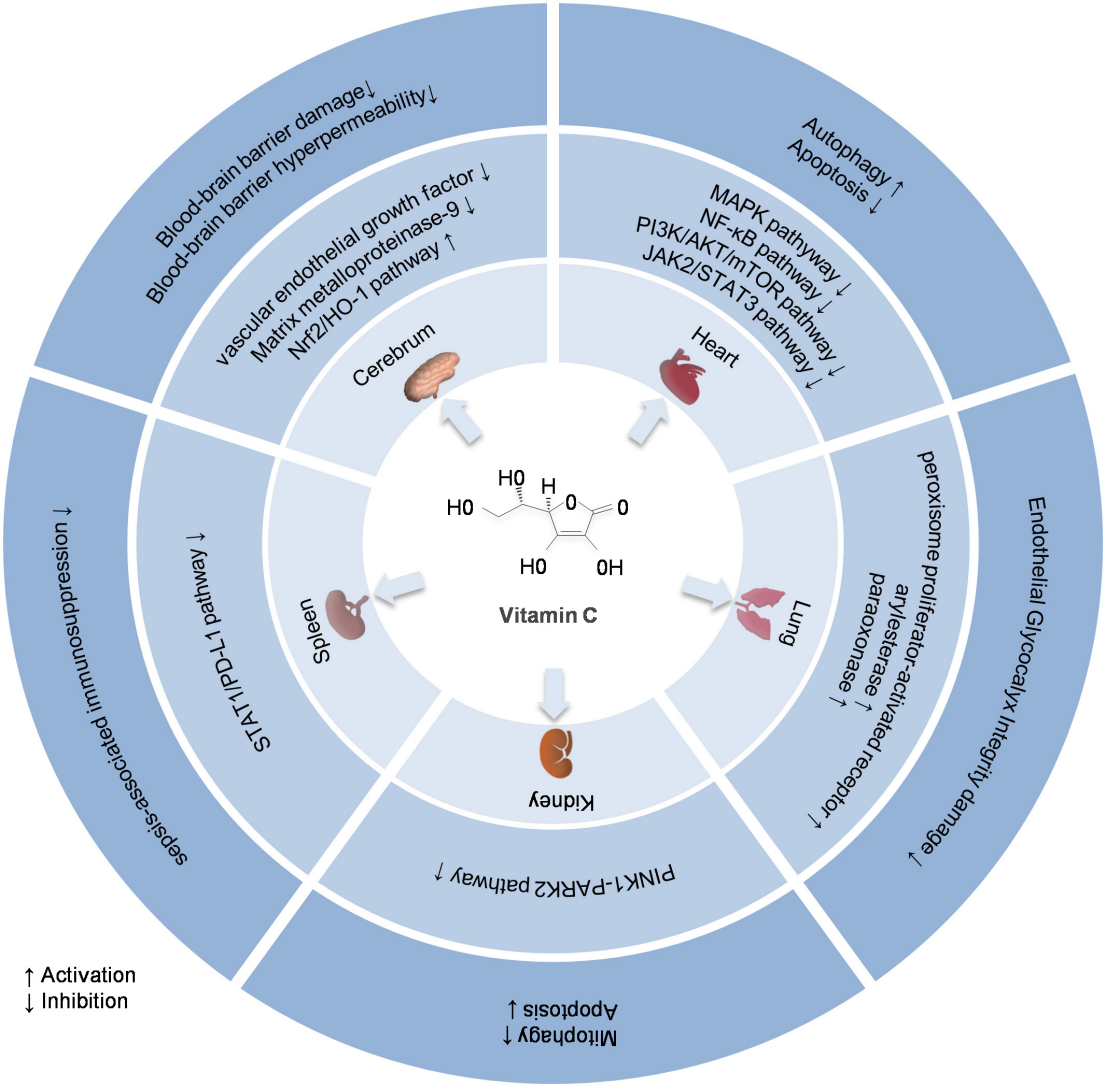
Molecular protective mechanisms of vitamin C in sepsis-induced organ injury. Nrf2/HO-1 pathway, Nuclear factor erythroid 2-related factor 2 and heme oxygenase-1 pathway; MAPK pathway, Mitogen-activated protein kinase pathway; NF-κB pathway, Nuclear factor kappa B pathway; PI3K/AKT/mTOR pathway, Phosphatidylinositol 3-kinase/protein kinase B/mammalian target of rapamycin pathway; JAK2/STAT3 pathway, Janus Kinase2/Signal Transducer and Activator of Transcription 3 pathway; PINK1-PARK2 pathway, PTEN-induced kinase 1-Parkin RBR E3 ubiquitin-protein ligase pathway; PD-1, Programmed death receptor-1.

In addition to its antioxidant and anti-inflammatory properties, vitamin C protects organs in sepsis by targeting different pathways. The following sections delineate the organ-protective mechanisms and therapeutic outcomes substantiated by experimental and clinical evidence. Figure 1 shows a comprehensive overview of the key molecular pathways involved.

### 2.1 Cardioprotective effects

Vitamin C protected against sepsis-induced cardiac injury through multiple pathways. First, it significantly reduced pro-inflammatory cytokine levels (interleukin-1β, interleukin-6, and tumor necrosis factor-α) in cardiac tissues while increased anti-inflammatory mediators (interleukin-10). This effect was mediated by suppressing phosphorylation in several critical signaling pathways, including P38, Erk1/2, and JNK in the mitogen-activated protein kinase pathway; nuclear factor kappa B and IKKα/β in the NF-κB pathway (20, 21); and the Janus Kinase 2/Signal Transducer and Activator of Transcription 3 pathway (22). Simultaneously, it mitigated oxidative damage by markedly augmenting myocardial antioxidant enzyme activities (including catalase, glutathione, glutathione peroxidase, and superoxide dismutase) and decreasing levels of oxidative stress markers like myeloperoxidase and malondialdehyde (20, 23). Additionally, vitamin C enhanced cellular autophagy through inhibition of the phosphatidylinositol 3-kinase/protein kinase B/mammalian target of rapamycin pathway, evidenced by increased Beclin-1 expression, an elevated LC3-II/LC3-I ratio, and reduced P62 expression in myocardial tissue, while concurrently suppressed apoptotic processes (21). Finally, vitamin C alleviated myocardial injury by downregulating NADPH oxidase 4 expression in cardiomyocytes, which diminished reactive oxygen species (ROS) production; this inhibition of ROS subsequently blocked activation of the ROS-protein kinase B/mammalian target of rapamycin pathway, ultimately suppressing pyroptosis (24). Clinically, 3 g/day vitamin C reduced the levels of cardiac injury markers (e.g., troponin I and B-type natriuretic peptide) in sepsis patients (25).

### 2.2 Neuroprotective effects

The hippocampus, essential for learning and memory, is vulnerable to inflammation, which directly suppresses long-term potentiation and impairs cognition (26). Moreover, sepsis-induced endothelial glycocalyx degradation releases heparan sulfate fragments that cross the blood-brain barrier and accumulate in the hippocampus to impair long-term potentiation by inhibiting the brain-derived neurotrophic factor/tropomyosin receptor kinase B signaling pathway (27). Critically, inflammatory mediators suppress expression of the sodium-dependent vitamin C transporter 2, thereby reducing neuronal vitamin C uptake (28) and leading to a sharp decline in brain vitamin C levels (29). Supplementation with vitamin C reduced the levels of proinflammatory cytokines, activated the nuclear factor erythroid 2-related factor 2 and heme oxygenase-1 pathways to counteract oxidative stress, and mitigated damage to the blood-brain barrier caused by matrix metalloproteinase-9 (endothelial glycocalyx-degrading enzyme). These effects significantly attenuated sepsis-induced pathological alterations, particularly CA1 pyramidal neuron loss, ultimately improving spatial learning and recognition memory (30, 31). Additionally, by inhibiting sepsis-induced overexpression of vascular endothelial growth factor and the resulting increase in vascular permeability, sodium ascorbate reduced cerebral edema, thereby restoring cerebral perfusion and rapidly reversing the cerebral ischemia-hypoxia (32).

### 2.3 Pulmonary protection

The pulmonary endothelial glycocalyx, which is significantly thicker than in other microvascular beds, is critically involved in sepsis-induced acute lung injury (33). Its early degradation correlates with injury severity (34). For example, tumor necrosis factor-α activates heparanase to degrade heparan sulfate, a process that significantly reduces glycocalyx thickness (33). This is reflected clinically by a 23-fold increase in circulating heparan sulfate among patients with sepsis-induced lung injury compared to healthy controls (35) and higher syndecan-1 levels, another marker of glycocalyx degradation, in septic shock patients with acute respiratory distress syndrome (ARDS) compared with those without it (36).

Inhibiting the inflammatory response and protecting the endothelial glycocalyx layer constitute a pivotal therapeutic strategy against sepsis-induced lung injury. Studies found that vitamin C effectively attenuated lung inflammatory responses (e.g., by increasing peroxisome proliferator-activated receptor levels) and oxidative stress (e.g., by increasing the levels of arylesterase and paraoxonase) in a septic mouse model (37, 38). Clinically, vitamin C-treated patients with sepsis-associated ARDS exhibited attenuated syndecan-1 elevation and concurrent improvement in PaO_2_/FiO_2_ ratios at 48 h post-intervention. Specifically, for every 1 ng/ml reduction in syndecan-1 levels, the PaO_2_/FiO_2_ ratio increased by 8.85 (95% CI: 0.19–17.50; *P* = 0.045), suggesting that vitamin C improves lung function partly through endothelial glycocalyx preservation (39, 40). This effect on PaO_2_/FiO_2_ enhancement was also observed in critically ill COVID-19 patients (41). Furthermore, early vitamin C administration reduced the incidence of ARDS and ventilator-associated pneumonia in sepsis patients requiring mechanical ventilation (42).

### 2.4 Splenic immunomodulation

The septic mouse model induced significant pathological alterations in the spleen, manifested by enhanced cellular apoptosis, necrotic manifestations, and structural disorganization, accompanied by a substantial reduction in CD3+ T lymphocytes (43). During sepsis progression, the elevated expression of programmed death receptor-1 and its ligand suppressed T lymphocyte activation and induced T-cell exhaustion, resulting in a significant immunosuppressive phenotype (44, 45). Vitamin C intervention downregulated programmed death receptor-1 expression levels by inhibiting the phosphorylation of signal transducer and activator of transcription 1 at the Y701 site, thereby reducing immune cell apoptosis and improving pathological changes in the spleen, ultimately reversing the sepsis-induced immunosuppression (43).

### 2.5 Renal protective mechanisms

Sepsis frequently induces acute kidney injury, which occurs in approximately 50% of patients and confers a higher mortality than non-septic acute kidney injury (46–48). Accumulating evidence underscores a protective role for vitamin C. Knocking out sodium-dependent vitamin C transporter-1 and−2 blocks ascorbic acid uptake, increasing the susceptibility of renal tubular cells to lipopolysaccharide-induced caspase-3-dependent apoptosis. Conversely, vitamin C supplementation promoted PTEN-induced kinase 1-Parkin RBR E3 ubiquitin-protein ligase axis-mediated mitophagy and inhibited apoptosis in human renal tubular cells (49). Combined therapy with hydrocortisone and vitamin C, or the combination of hydrocortisone, vitamin C, and thiamine (HAT), mitigated renal pathological injury during sepsis by attenuating inflammatory responses and oxidative stress (50, 51). Additionally, vitamin C downregulated the expression of drug-resistance genes and biofilm-associated genes in uropathogenic Escherichia coli, suppressed bacterial proliferation, and ultimately diminished inflammatory cell infiltration coupled with histopathological lesions in bladder and renal tissues (52, 53). Clinically, a multicenter randomized controlled trial reported reduced need for renal replacement therapy with vitamin C supplementation (OR: 0.28, 95% CI: 0.078–1.0; *P* = 0.05) (54), and large observational datasets indicated that vitamin C reduced mortality in patients with sepsis-induced acute kidney injury (55). However, conflicting evidence exists. One cohort study reported an increased acute kidney injury risk among sepsis patients treated with vitamin C (adjusted OR: 1.61, 95% CI: 1.09–2.39; *P* = 0.02) (56), and a randomized controlled trial found higher renal replacement therapy rates—though the interpretability of the latter finding is compromised by a high baseline renal replacement therapy rate in the vitamin C group (57). Consequently, large-scale, high-quality randomized controlled trials are warranted to clarify the true efficacy of vitamin C in sepsis-associated kidney injury.

## 3 Restoration of microcirculatory dynamics by vitamin C

Microcirculatory dysfunction is closely associated with the progression of sepsis and does not always improve in parallel with the stabilization of macrocirculation (2, 58). Theoretically, vitamin C mitigates oxidative stress and inhibit the secretion of inflammatory mediators, which suppress the overexpression of glycocalyx degrading enzyme, maintain the structural integrity of endothelial glycocalyx (40, 59), thereby preserving microcirculatory function (60–62). Nevertheless, a clinical study of high-dose intravenous vitamin C in sepsis patients requiring norepinephrine found this effect did not persist long-term (63). Additionally, the nitric oxide produced by eNOS is a key mediator of vasodilation. A critical aspect of eNOS function involves BH4, an essential cofactor readily oxidized to dihydrobiopterin during sepsis. Vitamin C stabilizes BH4 by inhibiting this oxidation. This action reduces dihydrobiopterin binding to eNOS, decreases ROS production, and maintains BH4 bioavailability, thereby preserving eNOS catalytic function and nitric oxide generation (18, 64–66). Furthermore, vitamin C scavenges superoxide anion radicals, preventing their reaction with nitric oxide to form peroxynitrite (67–69). These effects were substantiated by clinical research. Lavillegrand et al. (70) reported that intravenous vitamin C administration significantly improved mottling scores and capillary refill times in septic shock patients. When forearm endothelium-dependent microvascular reactivity was assessed via acetylcholine challenge tests, a marked elevation in cutaneous microvascular blood flow within 1 h after vitamin C administration. Furthermore, Wang's research (71) demonstrated that, compared to hydrocortisone monotherapy, HAT therapy significantly improved Perfusion Vessel Density, Total Vessel Density, and Microvascular Flow Index in septic shock patients. These specific sublingual microcirculation parameters were shown by meta-analysis to be lower in non-survivors than survivors (72). Collectively, these findings support the therapeutic value of vitamin C in the microcirculation of septic shock patients.

## 4 Clinical outcomes: evidence and controversies

Clinical trials of vitamin C for sepsis have yielded inconsistent outcomes (Table 1). The CITRIS-ALI trial (2019) reported that high-dose vitamin C significantly reduced 28-day all-cause mortality in sepsis patients with ARDS and increased ICU-free days between days 28 and 60 (39). Subsequently, the ViCTOR (76) and ORANGES trials (77) employing HAT reported accelerated shock resolution, consistent with findings from vitamin C monotherapy studies (42, 75). However, other trials evaluating either vitamin C monotherapy or HAT observed no reduction in vasopressor duration (78–82). Among these studies, the ViCTOR and ORANGES trials initiated treatment early (within 6 and 12 h of diagnosis, respectively). In contrast, the study by Lyu et al. (80), while also employing early intervention, exclusively enrolled patients with established septic shock and used a relatively high vitamin C dose (8 g/day). The neutral outcomes reported by Lyu et al. (80) align with a meta-analysis suggesting that high-dose vitamin C (≥6 g/day or 100 mg/kg/day) does not facilitate shock reversal in patients with septic shock, though a benefit may be maintained for those with sepsis without shock at enrollment (83). This underscores the critical influence of treatment timing and dosing strategy, a point explored in the next section.

**Table 1:** Characteristics of recent randomized controlled trials of vitamin C monotherapy in sepsis.

**Author, year**	**Study type**	**Study population**	**Sample size**	**Patient's age (year)**		**Dosing regimen**	**Primary site of infection**		**SOFA score**	**Short-term mortality (vitamin C group vs. control group)**
			**Vitamin C group/ control group**	**Vitamin C group**	**Control group**		**Vitamin C group**	**Control group**		
Fowler, 2019 (39)	Multicenter, double- blind, placebo randomized controlled trial	Adult patients with sepsis and ARDS	84/83	54 (39, 67)^a^	57 (44, 70)	50 mg/kg every 6 h for 96 h	Thorax: 82.1%; Abdomen: 7.1%; Urinary tract: 3.6%; Central nervous system: 1.2%	Thorax: 69.9%; Abdomen: 15.7%; Urinary tract: 2.4%; Central nervous system: 3.6	Difference in SOFA score change at 96 h between groups:−0.10 (95% CI: −1.23 to 1.03); *P* = 0.86	29.8 vs. 46.3%; *P* = 0.03
Lamontagne, 2022 (73)	Multicenter, double- blind, placebo randomized controlled trial	Adult patients with proven or suspected infection and receiving a vasopressor	429/433	65.0 ±14.0^b^	65.2 ± 13.8	50 mg/kg every 6 h for 96 h	Pulmonary: 33.8%; Gastrointestinal or intra-abdominal: 31.0%; Blood: 12.8%; Skin or soft tissue: 12.8%; Urinary: 11.4%	Pulmonary: 36.7%; Gastrointestinal or intra-abdominal: 25.9%; Blood: 13.6%; Skin or soft tissue: 14.3%; Urinary: 12.7%	Difference in SOFA score at 96 h between groups: −0.03 (95% CI: −0.90 to 0.85); *P* >0.05	35.4 vs. 31.6%; *P* > 0.05
Rosengrave, 2022 (74)	Single-center, double- blind, placebo randomized controlled trial	Adult patients with septic shock	20/20	69 (64, 76)	66 (57, 71)	25 mg/kg every 6 h for 96 h	Abdominal: 40.0%; Pulmonary: 15.0%; Skin/soft tissue: 20.0%; Blood: 15.0%; Urinary tract: 10.0%	Abdominal: 30.0%; Pulmonary: 30.0%; Skin/soft tissue: 15.0%; Blood: 20.0%; Urinary tract: 5.0%	Difference in SOFA score at 96 h between groups: 1.2 (95% CI: −3.8 to 6.1); *P* = 0.64	30.0 vs. 35.0%; *P* > 0.05
El Driny 2022, (42)	Single-center, double- blind, placebo randomized controlled trial	Adult patients with sepsis and required mechanical ventilation within 24 h from ICU admission	20/20	53.0 ± 23.3	52.1 ± 18.8	1.5 g every 6 h for 96 h	Central venous catheter: 35.0%; Urinary tract infection: 25.0%; Abdominal: 20.0%; Skin and soft tissue: 10.0%	Central venous catheter: 30.0%; Urinary tract infection: 30.0%; Abdominal: 10.0%; Skin and soft tissue: 20.0%	Comparison of SOFA scores at 96 h: 5.2 ± 2.0 in the vitamin C group vs. 9.0 ± 2.6 in the control group; *P* < 0.01	15.0 vs. 45.0%; *P* = 0.04
Wacker, 2022 (57)	Multicenter, double-blind, randomized controlled trial	Adult patients with septic shock	60/64	68.9 (60.1, 79.9)	73.0 (60.8, 80.0)	1 g intravenous injection followed by 250 mg/h infusion for 96 h or 24 h vasopressor-free (whichever first)	Pulmonary: 21.6%; Urinary: 26.6%; Gastrointestinal/ biliary: 20%; Soft tissue/skin: 6.7%; Bacteremia: 10.0%	Pulmonary: 25.0%; Urinary: 21.9%; Gastrointestinal/ biliary: 21.9%; Soft tissue/skin: 10.9%; Bacteremia: 6.2%	No significant difference in SOFA score change between groups	26.7 vs. 40.6%; *P* = 0.1
Belousoviene, 2023 (63)	Single-center, double- blind, placebo randomized controlled trial	Adult patients with sepsis requiring norepinephrine	12/11	66 (56, 78)	60 (50, 76)	50 mg/kg every 6 h for 96 h	Pneumonia: 33.3%; Abdomen: 58.4%; Urinary tract: 8.3%	Pneumonia: 45.5%; Abdomen: 9.1% Urinary tract: 27.3%	Comparison of SOFA scores change at 96 h: 0 (0, 4) in the vitamin C group vs. 2 (0, 4) in the control group; *P* = 0.36	58.3 vs. 54.5%; *P* = 0.85
Li, 2024 (65)	Single-center, double-blind randomized controlled trial	Adult patients with septic shock	High-dose vitamin C group: 20; low-dose vitamin C group: 20 control group: 18	High-dose vitamin C group:72.00 ± 14.67; low-dose vitamin C group: 63.30 ± 17.13	67.78 ± 13.53	High-dose vitamin C (150 mg/kg/d) and low-dose vitamin C (50 mg/kg/d) for 96 h	High-dose vitamin C group: lungs: 40.0%; Biliary tract: 30.0%; Blood: 20.0%; Abdomen: 10.0% low-dose vitamin C group: lungs: 60.0%; Biliary tract: 10.0%; Blood: 10.0%; Abdomen: 20.0%	Lungs: 66.67%; Biliary tract: 11.1%; Blood: 11.1% Abdomen: 11.1%	Comparison of SOFA scores change at 96 h: −0.27 ± 0.24 in the high-dose vitamin C group vs. −0.46 ± 0.34 in the low-dose vitamin C group vs. −0.27 ± 0.22 in the control group; *P* > 0.05	High-dose vitamin C group: 0% vs. low-dose vitamin C group: 10.0% vs. control group: 16.7%; no significant pairwise differences
Jiang, 2024 (25)	Randomized controlled trial	Patients with sepsis and APACHE II Scores ≥12	42/41	53.25 ± 13.21	53.28 ± 13.18	3 g everyday for 72 h	None	None	Post-treatment SOFA scores significantly lower in the vitamin C group compared to control group; *P* < 0.05	9.52 vs. 29.27%; *P* < 0.05
Mishra, 2024 (75)	Randomized controlled trial	Adult patients with suspected infection, having temperature >38 °C, heart rate >100 beats per minute, and quick SOFA scores ≥2	25/25	None	None	2.5 g every 8 h for 5 days	Surgical patients	Surgical patients	Comparison of SOFA scores at 6 days: 3.91 ± 2.76 in the vitamin C group vs. 6.00 ± 3.64 in the control group; *P* = 0.04	12.0 vs. 24.0%; *P* = 0.52
Vandervelden, 2025 (54)	Multicenter, double-blind, randomized controlled trial	Adult patients with a suspected infection and a National Early Warning Scores ≥5	147/145	64.7 ± 16.2	67.0 ± 13.9	1.5 g every 6 h for 96 h	Respiratory: 46.3%; Gastro-intestinal: 7.5%; Urinary: 19.1%; Skin or soft tissue: 11.6%	Respiratory: 53.1%; Gastro-intestinal: 8.3%; Urinary: 23.5%; Skin or soft tissue: 6.9%	Comparison of the average post-baseline SOFA scores on day 2 to 5: 1.98 (95% CI: 1.69 to 2.32) in the vitamin C group vs. 2.19 (95% CI: 1.87 to 2.56) in the control group; *P* = 0.30	12.0 vs. 7.9%; *P* = 0.27

ARDS, Acute respiratory distress syndrome; APACHE, Acute physiology and chronic health evaluation; SOFA, Sequential organ failure assessment; CI, Confidence intervals.

^a^Median (interquartile range).

^b^Mean ± Standard deviation.

With the exception of the VITAMINS trial (82), which reported an unadjusted improvement in sequential organ failure assessment (SOFA) scores, other major randomized controlled trials have not shown mortality or SOFA benefits with HAT (76–80, 84, 85). The unclear efficacy of vitamin C monotherapy is compounded by its combination with corticosteroids and thiamine, complicating the evaluation of its independent role and synergy.

More concerningly, the LOVIT trial revealed not only lack of mortality benefit with high-dose vitamin C in septic shock but an increased risk of death or persistent organ dysfunction (73), with no differential effects across inflammatory phenotypes (86). This finding was reinforced by a Bayesian reanalysis indicating vitamin C significantly increased adverse outcomes (28-day mortality or organ dysfunction) in critically ill patients with confirmed/suspected infections requiring vasoactive medications (RR 1.20, 95% CI 1.04–1.39; 99% probability of harm) (87). Conversely, similar to the CITRIS-ALI approach, recent studies targeting precise populations (such as mechanically ventilated patients or those with acute kidney injury) found vitamin C reduced sepsis mortality risk (42, 55). These conflicting results indicate heterogeneous treatment responses to vitamin C among different sepsis phenotypes. Future studies should aim to delineate these subgroups, particularly since cohort data have already suggested survival differences stratified by infection site in patients receiving vitamin C (16).

## 5 Optimizing clinical applications: challenges and research priorities

**Figure 2:**
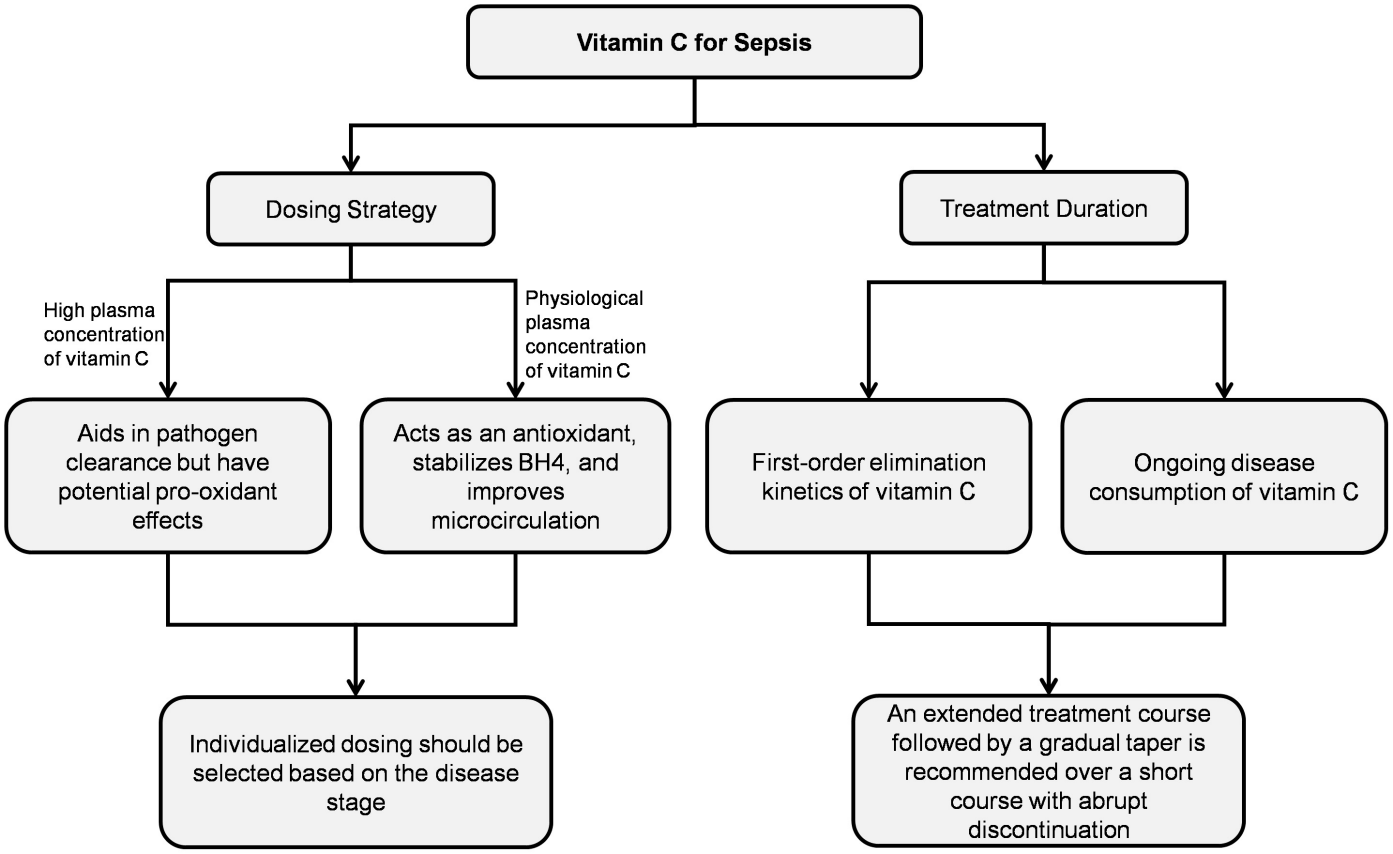
Vitamin C treatment regimens.

Animal studies have demonstrated that vitamin C exerts multiple protective effects against sepsis-induced organ damage, including antioxidant, anti-inflammatory, anti-apoptotic, and pro-autophagy. Furthermore, *in vitro* human studies found that vitamin C selectively inhibited lipopolysaccharide-induced cytokine secretion in a concentration-dependent manner (88–91). However, clinical trial results remain controversial. These inconsistencies suggest that the actual clinical efficacy of vitamin C in sepsis patients may be modulated by multiple factors (Figure 2).

### 5.1 Dosing strategies

The plasma concentration of vitamin C determines its distinct biological effects. Clinical studies on critical illnesses used wide dosing ranges (450–24,000 mg/day) (92), and high-dose short-course regimens represent the mainstream therapeutic strategy for vitamin C in sepsis-related research. However, subgroup data from the meta-analysis revealed that vitamin C at effective doses of 25–100 mg/kg/day significantly improved mortality in sepsis patients (OR: 0.80; 95% CI: 0.65–0.97; *P* = 0.03). No survival benefit was observed at doses exceeding 100 mg/kg/day, indicating the importance of dosage optimization in clinical decision-making (93).

#### 5.1.1 Effects at physiological concentrations

At physiological concentrations (1–100 μM), vitamin C chemically stabilized BH4, saturating at 100 μM to preserve eNOS function (18). Research has found that a single intravenous injection of 40 mg/kg vitamin C significantly improved skin microvascular blood flow in septic shock patients at 1 h (70). Administering 1.5 g per dose every 6 h significantly improved microcirculatory blood flow in shock patients within 24 h (71). However, when the plasma vitamin C concentration reached 1 mM, the BH4 concentration did not increase but instead slightly decreased. This effect was likely related to the pro-oxidant activity of high-concentration vitamin C interfering with BH4 levels (18).

#### 5.1.2 Implications of high-dose administration

High plasma concentrations of vitamin C contributed to pathogen clearance but may also carry potential adverse effects. Salmonella-derived glyoxalate inhibited host TET2 DNA dioxygenase, aiding bacterial antibiotic resistance. Research has demonstrated that administration of 400 mg/kg vitamin C suppresses glyoxalate production, reactivates TET2, and reverses glyoxalate-induced downregulation of pro-inflammatory genes (Nos2, Cxcl9, and Cxcl10) essential for infection defense (94). However, intravenous administration of 200 mg/kg/day vitamin C in sepsis patients could yield serum concentrations of 3,082 μM (95). At millimolar concentrations in extracellular fluids, ascorbic acid promoted H_2_O_2_ generation in a concentration-dependent manner (19). Although normal tissues efficiently decompose H_2_O_2_ due to abundant catalase and glutathione peroxidase, septic tissues exhibit reduced levels of these antioxidant enzymes (96–98) and accumulate elevated intracellular free iron ions (99, 100). Under these conditions, high-dose vitamin C may potentially exacerbate oxidative stress via the Fenton reaction. Additionally, neutrophils from sepsis patients showed reduced neutrophil extracellular trap formation at vitamin C concentrations ≤ 1 mM, whereas ≥5 mM concentrations enhanced chemotaxis, phagocytic activity, and neutrophil extracellular trap generation (101). Although neutrophil extracellular trap combat microbial infections, excessive neutrophil extracellular trap formation could exacerbate inflammatory responses, promote immunothrombosis, cause host tissue damage, and ultimately lead to adverse clinical outcomes (102–106). Such drug-induced damage cannot be overlooked in sepsis patients experiencing hyperinflammatory states.

#### 5.1.3 Translational gaps between preclinical and clinical studies

Most preclinical studies administer high-dose intravenous vitamin C within hours of sepsis model induction or preemptively (21, 23, 43, 64, 98), a timing that is clinically challenging to replicate due to the difficulty in promptly identifying sepsis in patients (107). However, this timing discrepancy proves critical, as demonstrated by one septic animal model where high-dose vitamin C administered 12 h post-modeling failed to achieve the therapeutic efficacy observed with immediate administration (64). Consequently, very early high-dose vitamin C administration in animal models effectively clear pathogens, thereby attenuating infection-induced excessive inflammation and oxidative stress. But its potential pro-oxidant effects may exacerbate oxidative stress in septic shock—a state characterized by ongoing depletion of already diminished antioxidant enzymes. This divergence was indirectly supported by meta-analysis findings: vitamin C at ≥6g/day or 100 mg/kg/day reduced mortality in sepsis patients without shock upon enrollment but not in those with established shock (83). Collectively, these findings indicate that the stage of sepsis is critical, and high-dose vitamin C may be unsuitable for patients with established shock.

### 5.2 Treatment duration considerations

#### 5.2.1 Pharmacokinetics of vitamin C

The lack of the L-gluconolactone oxidase gene in humans results in an inability to synthesize endogenous vitamin C and requires additional supplementation. Vitamin C exists *in vivo* in two biochemical forms: ascorbic acid and dehydroascorbic acid. Ascorbic acid undergoes oxidation to dehydroascorbic acid, whereas dehydroascorbic acid irreversibly degrades into oxalic acid that undergoes renal excretion via urinary pathways. Following high-dose intravenous administration, vitamin C was eliminated with first-order kinetics at a rapid rate. The elimination half-life is approximately 2 h in cancer patients, while in septic shock patients, it is approximately 6.9 h (108–110). This forms the basis for the every 6-h dosing schedule in many experimental protocols.

#### 5.2.2 Influence of treatment duration on efficacy

Recent survival curves in a septic mouse model demonstrated that consecutive 8-day vitamin C treatment significantly improved survival rates compared to the placebo group, while a shorter 4-day regimen showed no beneficial effect (111). This is consistent with a cohort study (16) in which sepsis patients receiving prolonged therapy (≥5 days, mean daily dose ~6 g) achieved superior clinical outcomes compared to those with shorter durations (1–2 days or 3–4 days), with significantly reduced in-hospital mortality and 90-day mortality. However, most randomized controlled trials investigating vitamin C for sepsis employed treatment durations of 3–7 days, predominantly ≤ 4 days (91). It should be noted that 15% of patients with multiple organ dysfunction receiving vitamin C therapy (2 g/day or 10 g/day) exhibited serum concentrations dropping to deficiency levels within 48 h post-treatment discontinuation (112). Analysis of the LOVIT trial, which used a 4-day treatment course, revealed no mortality difference during the treatment period itself; however, the vitamin C group experienced significantly more deaths within 1 week after treatment cessation (RR: 1.9, 95% CI: 1.2–2.9; *P* =0.004) (113). This may be attributable to the rapid decline in serum vitamin C concentration and resultant redox imbalance following abrupt discontinuation. A recent study found that sepsis patients with baseline SOFA scores ≥6 receiving 6 g/day vitamin C for 4 days showed significantly lower SOFA scores than controls, though with no significant mortality difference (54). In comparison, another study using the same dose (6 g/day) demonstrated a 46% reduction in mortality among sepsis patients with SOFA scores ≥9 when vitamin C supplementation was maintained throughout their ICU stay (15). These findings underscore the significance of determining an appropriate duration of vitamin C therapy in sepsis patients to avert rebound adverse effects associated with abrupt treatment termination.

### 5.3 Identification of responsive patient populations

Researchers have classified sepsis into four distinct phenotypes: the α phenotype, characterized by minimal vasopressor requirements; the β phenotype, typically observed in older patients with multiple underlying chronic conditions and renal insufficiency; the γ phenotype, predominantly exhibiting inflammatory responses and pulmonary dysfunction; and the δ phenotype, more frequently associated with hepatic insufficiency and septic shock. Mortality rates and inflammatory responses varied across these phenotypes, underscoring the heterogeneity of sepsis (114). Vitamin C primarily exerts antioxidant and anti-inflammatory effects. Observational studies revealed that patients receiving vitamin C therapy derive significantly greater benefit if they belong to a high-inflammatory response group compared to a low-inflammatory response group (115, 116). Moreover, an *in vitro* study demonstrated that vitamin C suppressed the release of inflammatory cytokines and ROS in human whole blood induced by Escherichia coli, but not by Staphylococcus aureus (117). This suggests a differential effect of vitamin C treatment depending on the type of sepsis, although further confirmation through randomized controlled trials is required.

## 6 Conclusion

In conclusion, vitamin C represents a promising adjunctive therapy in sepsis, conferring organ protection through multiple mechanisms. However, its complex pharmacokinetics and the heterogeneity of sepsis necessitate optimized timing and individualized regimens. As demonstrating its universal mortality benefit is challenging, future efforts should prioritize identifying patient subgroups with a favorable response to vitamin C. A proper assessment of its utility should integrate a spectrum of measures that encompass both direct biological measures, such as oxidative stress markers (e.g., total oxidant status, total antioxidant status) and inflammatory markers (e.g., Interleukin-6), and indirect functional parameters, including microcirculatory hemodynamics and organ function scores, to comprehensively evaluate therapeutic efficacy.
